# Mastoiditis Now Affects Adults: A Case Report of an Occurrence of the Disease in an 82-Year-Old Male

**DOI:** 10.7759/cureus.53794

**Published:** 2024-02-07

**Authors:** Nao Nakasato, Latha Ganti

**Affiliations:** 1 Biology, United World College, Montezuma, USA; 2 Medical Sciences, The Warren Alpert Medical School of Brown University, Providence, USA; 3 Emergency Medicine and Neurology, University of Central Florida College of Medicine, Orlando, USA

**Keywords:** streptococcal infection, hemophilus influenzae, acute otitis media, mastoiditis, complications of acute mastoiditis

## Abstract

Mastoiditis is typically considered a suppurative complication of otitis media seen in children. Vaccines and therapeutics can change the demographics of diseases. Childhood vaccination against Haemophilus influenza, for example, has shifted the mean age for ear, nose, and throat infections caused by this bug to age 25, whereas this used to be most prevalent in the pediatric age group previously. The authors present the case of an 82-year-old man who had mastoiditis. This case serves as a reminder to avoid anchoring bias when seeing an undifferentiated patient in the emergency department.

## Introduction

Mastoiditis, a potentially serious bacterial infection affecting the mastoid bone, is a condition of significant concern in clinical practice. Despite advancements in antibiotic therapy, it remains a challenging entity due to its varied presentations and potential for serious complications. The authors present the case of an 82-year-old male with mastoiditis. This is somewhat unusual as most of the literature reports on mastoiditis in children. It was also diagnostically challenging as the patient initially complained of only generalized weakness. The workup included CT imaging, which helped to make the diagnosis. This case reminds us to keep our differential broad and physical examination complete when evaluating a patient with a vague complaint such as weakness. It is important to note that in part due to routine antibiotics for otitis media, mastoiditis needs to remain in the differential, especially in patients who cannot give a proper history. The importance of physical examination cannot be over-emphasized. Adults don't usually get bacterial ear infections, and the elderly may have difficulty communicating because of sensory impairments and disabilities. 

The mastoid process is the hardest part of the temporal bone and it is connected to the middle ear space via contiguous air cells. Mastoiditis typically arises as a complication of acute otitis media (AOM); it arises in the middle ear and spreads locally to the mastoid triggering mucosal swelling, bone breakdown, and inflammatory responses that culminate in coalescent infection. 

Although less common, chronic mastoiditis [1] can occur due to unmitigated persistent inflammation, which may be seen most often in the setting of chronic otitis media. It is characterized by mastoid bone erosion and may be associated with cholesteatoma formation.

The classic triad of acute mastoiditis is postauricular redness, swelling, and tenderness. Sourcing the triad remains an important task for the clinician. However, important differential diagnoses exist. A systematic review of 35 studies found that the most common differentials in children were Langerhans cell histiocytosis, followed by rhabdomyosarcoma and acute myelogenous leukemia. The most common differentials in adults were squamous cell carcinoma, nasopharyngeal carcinoma, and histiocytosis of the Langerhans cells [2].

## Case presentation

An 82-year-old male with a history of dementia was brought into the ED for generalized weakness. The patient had previously complained of left-sided ear pain to his family. He had also experienced a fever at home. His history was significant for an ear infection a few weeks prior. The family was concerned because he seemed off balance sometimes and complained of ear pain. He did not have any fever in the ED, and the remainder of his vital signs were pulse: 81 beats per minute, respirations: 18 breaths per minute, and blood pressure: 153/81 mmHg.

**Figure 1:**
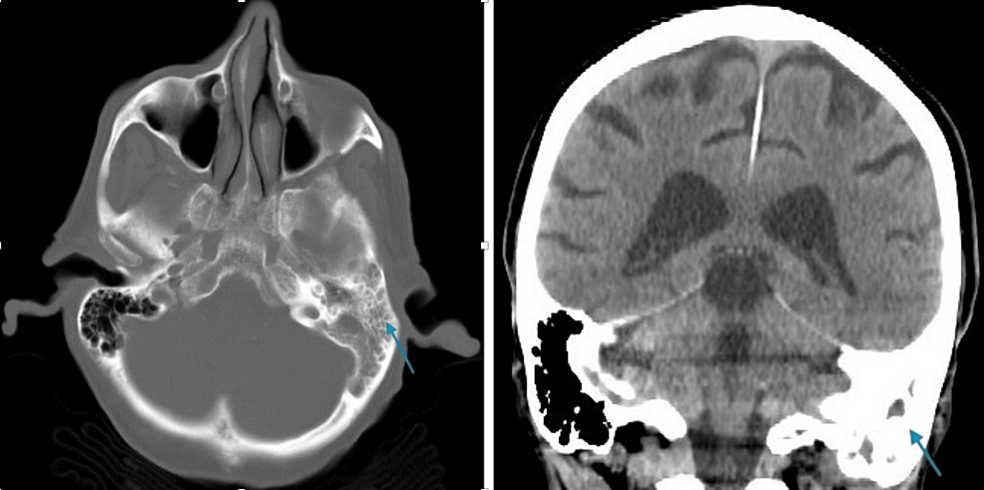
Noncontrast CT scan axial (left panel) and coronal (right panel) views demonstrating significant opacification of the mastoid air cells (blue arrow).

He could hear well in his ears, and the cranial nerve exam was unremarkable. An examination of the ears did not reveal any tympanic membrane bulging, effusion, or hemotympanum. There was a moderate amount of cerumen on the left but not enough to occlude the external auditory canal. There was some mild tenderness on palpation of the temporal scalp, but not of the mastoid. There was no erythema of the mastoid process. Given the patient’s baseline dementia, a non-contrast brain CT scan was obtained and revealed left-sided mastoiditis (Figure 1). The patient was admitted for intravenous antibiotics and an ENT consultation. He was discharged from the hospital on day three.

## Discussion

A CT scan is the initial investigation of choice for acute mastoiditis and was used to make the diagnosis for our patient. Partial-to-complete opacification of the mastoid air cells is classic, although non-specific. When there is an erosion of the lateral wall of the mastoid, a subperiosteal abscess is suspected. When there is an erosion of the sigmoid plate, an epidural abscess is suspected [3]. Neither were present in our patient. An MRI will also show partial-to-complete opacification of the mastoid air cells. Typical findings in mastoiditis include low signal on T1 images, high signal on T2 images, diffusion restriction on diffusion-weighted imaging/apparent diffusion coefficient (DWI/ADC) sequences, and mucosal contrast enhancement on T1 gadolinium-enhanced images [4]. It is important to note that fluid signal intensity in the mastoid should not be interpreted as mastoiditis without other evidence, such as mucosal contrast enhancement and/or diffusion restriction [5]. In a study of 23 patients, 87% had mastoiditis, and 12 (52%) of 23 patients had a subperiosteal abscess in addition to mastoiditis. MRIs identified subperiosteal abscesses and mastoiditis in all cases, with sensitivity and specificity for mastoiditis being 100% and 66%, respectively, and sensitivity and specificity for subperiosteal abscesses being both 100% [5].

Mastoiditis is classified into three categories according to the mechanism of infection: incipient mastoiditis, acute coalescent mastoiditis, and subacute mastoiditis. The most common presentation is acute coalescent mastoiditis, in which the epithelial lining is inflamed with erosion through bony septa of the mastoid air cells that are covered by epithelium and continuous with the middle ear cavity [6]. As mastoiditis is a complication of otitis media, the causative organisms include Group A beta-hemolytic streptococci, Staphylococcus aureus, Streptococcus pyogenes, and Haemophilus [7]. As children and infants are more susceptible to middle ear infections than adults, the majority of patients are under two years old, although it can occur at any age. With the advent of antibiotics, mastoidectomy, and PCV-7 (pneumococcal conjugate vaccine), the incidence of severe intracranial complication has decreased drastically: 0.002% of children with acute otitis media progress to acute coalescent mastoiditis with a mortality rate of less than 0.00001% [8,9]. However, if left untreated, it can lead to life-threatening sequelae, such as meningitis, intracranial abscess, and venous sinus thrombosis. Even with current management, the mortality of mastoiditis sequela remains high [5] and thus should be in the emergency medicine differential for ear pain. Antibiotics are the mainstay of initial treatment. Surgical intervention is considered in cases unresponsive to medical therapy, in the presence of complications, or when chronic disease with cholesteatoma is identified. Myringotomy with or without tube placement may be performed for middle ear decompression.

Mastoidectomy, either simple or radical, depending on the extent of the disease, is performed to remove infected mastoid air cells and drain deep or persistent abscess collections. In cases with cholesteatoma, tympanomastoid surgery is necessary to remove the cholesteatoma and reconstruct the middle ear.

## Conclusions

This case demonstrates the importance of considering mastoiditis in the differential diagnosis of otitis, even when a patient is in an atypical population for having this diagnosis. The advent of vaccines against traditional pediatric infections has resulted in a change in infection patterns, which has contributed to the occurrence and reporting of more cases of mastoiditis in adults.
